# Hypertrophic cardiomyopathy in cats: clinical prevalence, risk factors, and outcomes of arterial thromboembolism in a referral population in Tehran, Iran (2020–2024)

**DOI:** 10.1186/s12917-026-05428-5

**Published:** 2026-03-26

**Authors:** Mohammad Jalilian, Niusha Pahlevaninezhad, Zahrasadat Yazdanjoo, Mehrnoosh Naderizade, Roxanna Sarabandi, Alireza Motahari, Mehdi Mashayekh, Mohammad Jokar, Arman Abdous

**Affiliations:** 1 Department of Clinical Sciences, Faculty of Veterinary Medicine, Islamic Azad University, Garmsar, Ga.C Iran; 2 Department of Clinical Sciences, Faculty of Specialized Veterinary Sciences, Science and Research Branch, Islamic Azad University, Tehran, Iran; 3 College of Veterinary Medicine, Zabol Branch, Zabol, Sistan and Baluchestan Iran; 4 College of Veterinary Medicine, Shiraz University, Shiraz, Iran; 5 Faculty of Veterinary Medicine, University of Calgary, Calgary, AB T2N 1N4 Canada; 6 Faculty of Veterinary Medicine, Karaj Branch, Islamic Azad University, Karaj, Alborz Province Iran; 7 School of Public Health, University of Tehran, Tehran, Iran

**Keywords:** Feline hypertrophic cardiomyopathy, Arterial thromboembolism, Breed prevalence, Risk factors, Iran

## Abstract

**Background:**

Hypertrophic cardiomyopathy (HCM) is the most common cardiac disease in cats, yet epidemiological data from Iran are scarce. This study investigated the prevalence of HCM in a referral population of cats in Tehran, Iran, evaluated breed predisposition, characterized clinical findings, and assessed thromboembolic complications in cats referred for suspected cardiac disease.

**Methods:**

A retrospective multicenter study was conducted using medical records from seven major veterinary hospitals in Tehran between 2020 and 2024. A total of 7,013 cats evaluated for suspected cardiac disease were included. HCM was diagnosed by echocardiographic evidence of diastolic left ventricular wall thickness > 6 mm, excluding cats with documented or clinically confirmed systemic hypertension or hyperthyroidism. Clinical and diagnostic variables were extracted. Arterial thromboembolism (ATE) was diagnosed based on clinical presentation and Doppler ultrasonography. Univariate logistic regression was performed within the referral cohort to identify factors associated with HCM diagnosis, and Kaplan–Meier analysis was used to assess survival in cats with ATE.

**Results:**

HCM was diagnosed in 638 cats, yielding a clinical prevalence of 9.1% within the referral cohort. The highest within-breed prevalence of HCM in the referral cohort was observed in Persian cats (54.8%), followed by Sphynx (46.9%) and British Shorthair (45.0%).Univariate logistic regression analysis (*N* = 7,013) identified adult age (OR = 5.70, *p* < 0.001) and high body condition score (OR = 4.05, *p* < 0.001) as significant predictors of HCM. ATE occurred in 36 cats and carried a poor prognosis; only three cats survived after surgical intervention. Kaplan–Meier analysis demonstrated declining survival probability in cats with arterial thromboembolism over the available follow-up period.

**Conclusions:**

HCM is a frequent and clinically significant disease among cats referred for suspected cardiac disease to veterinary hospitals in Tehran, with clear breed predisposition and association with modifiable risk factors. Early screening may facilitate earlier detection and clinical management.

## Introduction

Hypertrophic cardiomyopathy (HCM) is recognized as the most prevalent cardiac disease in domestic cats and represents a major cause of morbidity and mortality worldwide [1, 2]. It is characterized by concentric thickening of the left ventricular myocardium in the absence of concurrent systemic conditions such as hypertension or hyperthyroidism [3]. HCM demonstrates a wide clinical spectrum, ranging from asymptomatic cases to those presenting with congestive heart failure or severe complications such as arterial thromboembolism (ATE) [4]. Clinically affected cats commonly exhibit respiratory distress, exercise intolerance, syncope, and acute hindlimb paresis associated with thromboembolic events [5, 6].

Advances in diagnostic imaging, particularly the widespread application of echocardiography, have enabled earlier and more accurate detection of HCM, thereby supporting improved therapeutic decision-making [7–9]. Studies conducted in North America and Europe have reported HCM prevalence rates of 10% to 15% in referral populations. Higher prevalence has been documented in certain breeds, including Maine Coons and Ragdolls, indicating a genetic predisposition [10, 11]. Despite growing global recognition of feline HCM, data regarding its prevalence, breed predisposition, clinical characteristics, and associated complications remain limited in many regions, including the Middle East.

In Tehran (Iran), where specific breeds such as Persian cats are highly prevalent, characterization of regional patterns of feline hypertrophic cardiomyopathy is essential for the development of appropriate screening and management strategies. Therefore, the objective of this study was to determine the prevalence of hypertrophic cardiomyopathy, describe associated clinical features and risk factors, and assess the frequency of thromboembolic complications in cats referred for suspected cardiac disease to major veterinary hospitals between 2020 and 2024.

## Methods

This multicenter retrospective observational study was based on a review of medical records from cats evaluated for suspected cardiac disease at seven major veterinary hospitals in Tehran, Iran, between January 2020 and December 2024. Eligible records were identified by reviewing hospital medical databases and selecting cases in which a cardiology assessment was performed due to clinical suspicion of cardiac disease. Presenting complaints and clinical findings suggestive of cardiac disease included dyspnea, open-mouth breathing, lethargy, syncope, and sudden hindlimb paresis.

A total of 7,398 feline medical records were initially identified across the seven hospitals during the study period. Of these, 385 cases were excluded, including 214 records with incomplete diagnostic data and 171 records with poor-quality echocardiographic images that did not allow reliable assessment. The final analyzed study population therefore comprised 7,013 cats evaluated for suspected cardiac disease across the seven hospitals. Cats were included only if they underwent cardiology assessment based on clinical suspicion arising from presenting complaints, physical examination findings, or referral indication. Cats with incidental echocardiographic findings identified during evaluation for unrelated conditions were not included.

Diagnostic evaluation typically included physical examination, electrocardiography (ECG), thoracic radiography, and transthoracic echocardiography. The availability and completeness of these assessments depended on the information recorded in the clinical files. Body condition score (BCS) was assessed during physical examination using a standardized 9-point scale, in which a score of 1 indicated emaciation and a score of 9 indicated severe obesity, in accordance with established feline clinical assessment methods and recent veterinary literature [12]. For statistical analysis, BCS was categorized such that a score of 5 out of 9 was considered ideal, while scores of 6 out of 9 or higher were classified as high BCS, indicating overweight or obese status. These categories were used to evaluate the association between body condition and the risk of hypertrophic cardiomyopathy (HCM).

Cardiac auscultation was performed by veterinary cardiologists certified by the Iranian Board of Veterinary Specialties (IBVS), who assessed for gallop rhythms, muffled heart tones, and systolic murmurs graded on a scale from 1 to 6.

Electrocardiography was performed using standard six-lead configurations according to routine clinical practice at each participating center. Tracings were obtained with cats positioned in right lateral recumbency and with a minimum recording duration of 60 s. ECG recordings documented heart rate, rhythm, and arrhythmias, including sinus tachycardia, ventricular or supraventricular tachycardia, and premature complexes. All ECGs were interpreted by board-certified veterinary cardiologists.

Thoracic radiographs were obtained in at least two orthogonal views to assess cardiomegaly, pulmonary edema, pleural effusion, and ascites. Vertebral heart score (VHS) was calculated where applicable. Radiographic cardiomegaly was defined as a vertebral heart score of 7.8 or greater measured on lateral thoracic radiographs, in accordance with recent feline radiographic studies [13]. Radiographs were interpreted by five veterinary radiologists certified by the Iranian Board of Veterinary Specialties, each with expertise in diagnostic imaging.

Transthoracic echocardiography was performed using standardized protocols at all participating hospitals. Two-dimensional, M-mode, and spectral and color Doppler modalities were employed in accordance with established clinical practice guidelines [5]. Echocardiographic studies were performed and interpreted by five board-certified veterinary cardiologists across the seven hospitals. Examinations were conducted using phased-array or micro-convex transducers operating at frequencies typically ranging from 5 to 10 MHz, with final frequency selection adjusted according to patient size and acoustic window to optimize spatial resolution and image quality. Echocardiographic measurements included interventricular septal thickness, left ventricular free wall thickness, and left atrial size, assessed using the left atrial-to-aortic root ratio (LA: Ao). Additional evaluations included assessment for valvular insufficiency, pericardial effusion, and systolic anterior motion of the mitral valve when documented in the clinical report.

The echocardiographic diagnosis of hypertrophic cardiomyopathy was based on a diastolic left ventricular wall thickness exceeding 6 mm in the absence of systemic hypertension or hyperthyroidism. Medical records were reviewed to exclude other recognized causes of secondary myocardial hypertrophy or infiltrative myocardial disease, including acromegaly, suspected neoplastic myocardial infiltration such as lymphoma, and non-cardiac conditions associated with transient or secondary myocardial thickening, including clinically significant dehydration or hypovolemia, severe anemia, and advanced chronic kidney disease, when documented [5]. Cats with these conditions were not classified as having primary hypertrophic cardiomyopathy unless the contemporaneous echocardiographic report supported a diagnosis of primary disease, supported by findings such as left atrial enlargement or diastolic dysfunction. Wall thickness measurements between 5.5 and 6.0 mm were considered equivocal and were not classified as hypertrophic cardiomyopathy unless supported by additional echocardiographic abnormalities. Allometric scaling of left ventricular wall thickness to body size was not performed due to the retrospective design and incomplete availability of standardized body size measurements across centers. Radiographic and echocardiographic findings were based on contemporaneous clinical interpretations recorded at the time of the initial investigation.

Systolic blood pressure was measured in all cats using a Doppler ultrasonic device following standardized measurement procedures [14]. Cats were allowed an acclimation period prior to measurement, and an appropriately sized cuff was placed at heart level. At least five consecutive systolic blood pressure readings were obtained during each measurement session, with the highest and lowest values discarded and the mean of the remaining readings recorded. Systemic hypertension was defined as persistently elevated systolic blood pressure of 160 mmHg or greater or when a diagnosis of hypertension was documented in the medical record based on repeated measurements and clinical assessment. A single elevated systolic blood pressure measurement was not used alone to classify a cat as hypertensive because of the potential influence of situational white-coat effects. Serum total thyroxine concentrations were measured in all cases using chemiluminescent immunoassay performed at an external reference laboratory (IDEXX Laboratories).

Arterial thromboembolic events were diagnosed based on clinical criteria, including sudden hindlimb paralysis, absent femoral pulses, and cold extremities, with Doppler ultrasonography used for confirmation when feasible. Treatment protocols were recorded and included the administration of anticoagulants such as clopidogrel and heparin, thrombolytic agents, analgesia, supportive care, and surgical thrombectomy when performed. Outcomes, including survival time, recurrence of thromboembolism, and mortality resulting from natural death or euthanasia, were documented for all cases. Follow-up continued until death or the end of the study period.

Statistical analyses were conducted using R software (version 4.2.2; R Foundation for Statistical Computing, Vienna, Austria). Descriptive statistics, including means, medians, interquartile ranges, and standard deviations, were calculated for continuous variables, while frequencies and percentages were reported for categorical variables. Prevalence estimates were calculated with 95% confidence intervals using the Wilson method. Clinical and diagnostic variables in cats diagnosed with hypertrophic cardiomyopathy were summarized descriptively. Univariate logistic regression analysis was used to evaluate associations between candidate predictors (sex, age category, and body condition score category) and the diagnosis of hypertrophic cardiomyopathy in the referral cohort (*n* = 7,013). Odds ratios with 95% confidence intervals were reported. Kaplan–Meier survival analysis was performed for cats diagnosed with arterial thromboembolism. A two-tailed p-value of less than 0.05 was considered statistically significant for all analyses.

## Results

Between January 2020 and December 2024, a total of 7,013 cats evaluated for suspected cardiac disease were included in the final analysis across seven major veterinary hospitals in Tehran, Iran. Hypertrophic cardiomyopathy (HCM) was diagnosed in 638 cats, corresponding to a prevalence of 9.1% (95% CI: 8.4% to 9.8%).

Breed-specific prevalence demonstrated marked variability. Persian cats exhibited the highest prevalence, with 54.8% of 400 individuals affected. This was followed by Sphynx cats at 46.9%, British Shorthair cats at 45.0%, Domestic Shorthair cats at 44.0%, and Scottish Fold cats at 42.7%. Lower prevalence rates were observed in Himalayan cats (35.5%), Exotic Shorthair cats (31.7%), British Longhair cats (29.2%), Domestic Longhair cats (28.3%), and Siamese cats (25.0%) (Table 1).

**Table 1:** Breed distribution and prevalence of hypertrophic cardiomyopathy (HCM) among cats evaluated for suspected cardiac disease

Breed	Cats with HCM (*n*)	Total cats of that breed evaluated (*n*)	Prevalence of HCM within breed (%)
British Longhair	35	120	29.2
British Shorthair	81	180	45.0
Domestic Shorthair	66	150	44.0
Domestic Longhair	34	120	28.3
Exotic Shorthair	19	60	31.7
Himalayan	39	110	35.5
Persian	219	400	54.8
Scottish Fold	64	150	42.7
Siamese	20	80	25.0
Sphynx	61	130	46.9

Prevalence represents the proportion of cats diagnosed with HCM within each breed among cats evaluated for suspected cardiac disease. Cats not diagnosed with HCM may have had other cardiac or non-cardiac conditions and were not considered healthy controls

On cardiac auscultation, muffled heart sounds were detected in 225 cats (35.3%), gallop rhythms in 87 cats (13.6%), and grade III systolic murmurs in 126 cats (19.7%). Electrocardiographic abnormalities were frequently observed, with atrial premature complexes identified in 197 cats (30.9%) and ventricular premature complexes in 151 cats (23.7%). Radiographic evaluation demonstrated cardiomegaly in 520 cats (81.5%), pulmonary edema in 150 cats (23.5%), and pleural effusion in 75 cats (11.8%). Echocardiographic assessment confirmed symmetric concentric left ventricular hypertrophy in all cases, isolated interventricular septal hypertrophy in 328 cats (51.4%), and left atrial enlargement in 110 cats (17.2%). Measurement of systolic blood pressure indicated that 540 cats (84.6%) had normal readings, while 98 cats (15.4%) had a single-visit systolic blood pressure measurement greater than 160 mmHg at presentation (Table 2).

**Table 2:** Clinical and diagnostic features in cats with hypertrophic cardiomyopathy (HCM) (*n* = 638)

Domain	Finding	*n* (%) (95% CI)
Auscultation	Normal	–
	Gallop	87 (13.6%; 11.1–16.6)
	Muffled heart sounds	225 (35.3%; 31.9–38.9)
	Murmur I	22 (3.4%; 2.2–5.2)
	Murmur II	116 (18.2%; 15.4–21.4)
	Murmur III	126 (19.7%; 16.9–22.9)
	Murmur IV	62 (9.7%; 7.7–12.1)
Electrocardiography (ECG)	Normal	290 (45.5%; 41.8–49.3)
	Atrial premature complexes (APCs)	197 (30.9%; 27.7–34.4)
	Ventricular premature complexes (VPCs)	151 (23.7%; 20.7–26.9)
Radiography	Normal	118 (18.5%; 15.7–21.7)
	Cardiomegaly	520 (81.5%; 78.3–84.3)
	Pulmonary edema	150 (23.5%; 20.5–26.8)
	Pleural effusion	75 (11.8%; 9.4–14.7)
Echocardiography	Symmetric concentric LV hypertrophy (IVSd and/or LVPWd > 6 mm)	638 (100.0%; 99.4–100.0)
	Isolated interventricular septal hypertrophy	328 (51.4%; 47.5–55.3)
	Left atrial enlargement (as reported clinically)	110 (17.2%; 14.4–20.4)
Blood pressure	SBP < 160 mmHg	540 (84.6%; 81.8–87.0)
	Single-visit SBP ≥ 160 mmHg	98 (15.4%; 13.0–18.2)

Findings are reported descriptively for cats with hypertrophic cardiomyopathy in the referral cohort. All cats met criteria for symmetric concentric left ventricular hypertrophy, with isolated interventricular septal predominance documented in a subset. Systolic blood pressure values reflect measurements at presentation; single elevated readings (SBP ≥ 160 mmHg) were not considered diagnostic of systemic hypertension and did not exclude cats from primary HCM classification. Numeric echocardiographic measurements were not consistently available and were therefore not summarized as continuous variables

*Abbreviations:* *HCM* Hypertrophic cardiomyopathy, *ECG* Electrocardiography, *APCs* Atrial premature complexes, *VPCs* Ventricular premature complexes, *IVSd* Interventricular septal thickness in diastole, *LVPWd* Left ventricular posterior wall thickness in diastole, *CI* Confidence interval

Univariate logistic regression analysis performed in the referral cohort (*n* = 7,013) identified age category and body condition score (BCS) as factors associated with HCM diagnosis. Adult cats had significantly higher odds of HCM diagnosis than senior cats (OR = 5.70; 95% CI: 3.18–10.23; *p* < 0.001). Cats with high BCS also had higher odds of HCM diagnosis than cats with ideal BCS (OR = 4.05; 95% CI: 2.29–7.17; *p* < 0.001). Sex was not significantly associated with HCM diagnosis in univariate logistic regression (*p* = 0.137); although males represented a higher proportion of HCM cases in this referral cohort (66.1%), this reflects the sex distribution of cats evaluated for suspected cardiac disease (Table 3).

**Table 3:** Factors associated with hypertrophic cardiomyopathy (HCM) diagnosis in the referral cohort (*N* = 7,013)

Related factor	Category	HCM cases (*n* = 638)	Odds ratio (95% CI)	*P*-value
Sex	Female	216	–	–
	Male	422	0.75 (0.47–1.20)	0.137
Age	Senior (> 9 years)	95	–	–
	Adult (5–9 years)	543	5.70 (3.18–10.23)	< 0.001*
Body condition score	Ideal (5/9)	112	–	–
	High (≥ 6/9)	526	4.05 (2.29–7.17)	< 0.001*

Odds ratios and P-values are derived from univariate logistic regression with hypertrophic cardiomyopathy diagnosis (yes/no) as the outcome in the referral cohort (*N* = 7,013); odds ratios are expressed relative to the stated reference categories, which are indicated by “–”

*Abbreviations:* *HCM* Hypertrophic cardiomyopathy, *BCS* Body condition score, *CI* Confidence interval

**Fig. 1:**
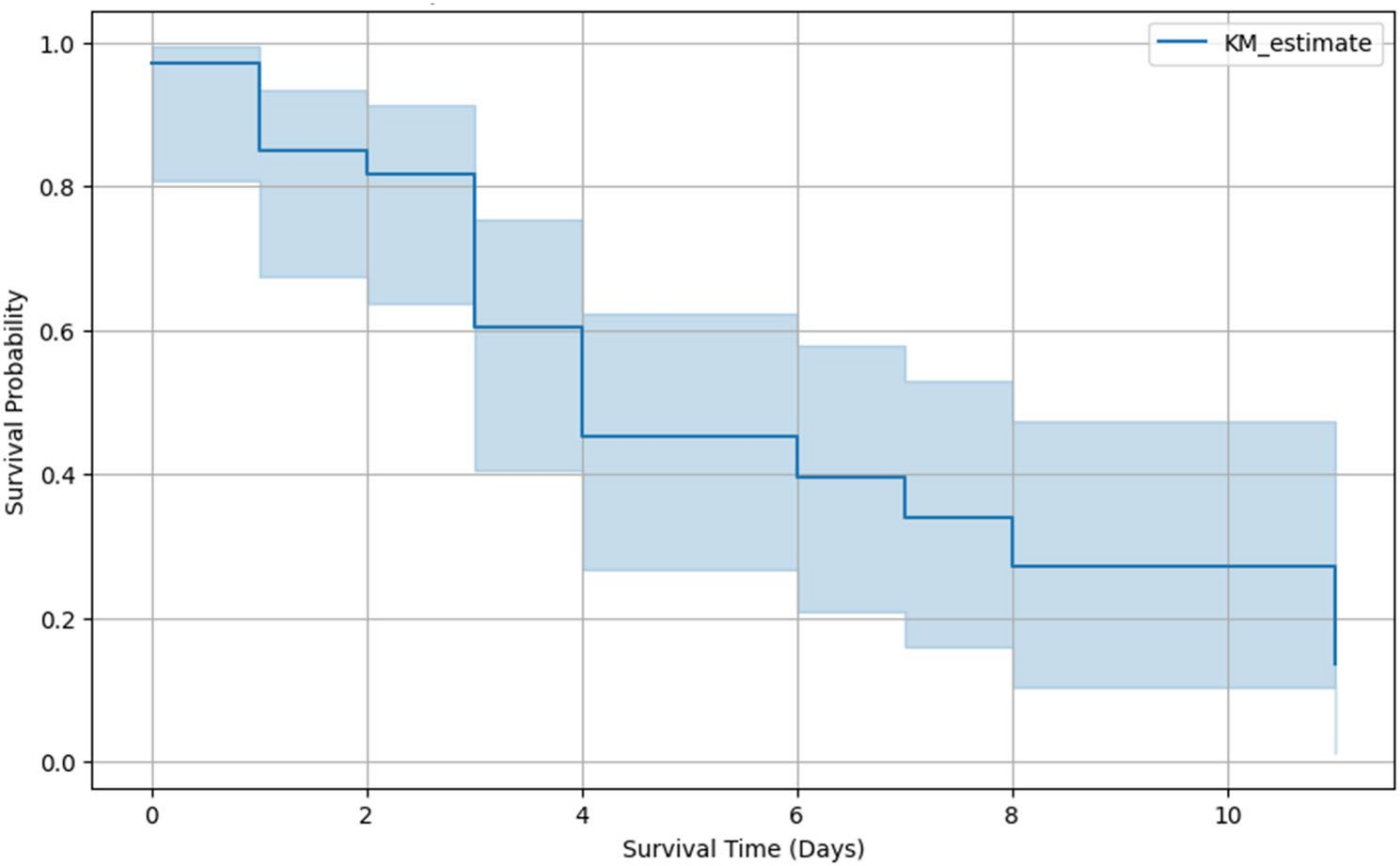
Kaplan-Meier survival curve for cats diagnosed with arterial thromboembolism (ATE). Survival probability declined progressively over time, from above 0.8 initially to approximately 0.2 by the final stage

Arterial thromboembolism was identified in 36 cats with hypertrophic cardiomyopathy and was associated with poor outcomes. Breed-specific distributions are summarized in Table 4, and Kaplan–Meier survival analysis demonstrated declining survival probability over the follow-up period (Fig. 1).

**Table 4:** Outcomes of thromboembolic complications in cats with hypertrophic cardiomyopathy

Disease	Breed	Total cases	Surgery	Euthanasia	Natural deaths
HCM	DSH	17	1 (died)	7	9
	Persian	9	1 (survived)	4	4
	DLH	3	Not performed	3	0
	British Shorthair	5	1 (survived)	1	3
	Scottish Fold	2	1 (survived)	0	1

*Abbreviations:* *HCM* Hypertrophic cardiomyopathy, *DSH* Domestic Shorthair, *DLH* Domestic Longhair

## Discussion

This study represents one of the most comprehensive retrospective investigations of hypertrophic cardiomyopathy in cats referred for suspected cardiac disease in Tehran, Iran, and provides valuable epidemiological insight into breed predispositions, clinical features, and associated complications. The observed prevalence of hypertrophic cardiomyopathy in the present cohort was 9.1%, reflecting clinical prevalence within a referral population of cats evaluated for suspected cardiac disease. This estimate is comparable to prevalence values reported in previous studies of feline cardiomyopathy conducted using different study designs and populations across Europe and North America [15–17]. Importantly, because the study population consisted exclusively of cats referred for suspected cardiac disease, the reported prevalence likely underestimates the true burden of hypertrophic cardiomyopathy in the general feline population, in which a substantial proportion of affected cats may remain asymptomatic or subclinical. In contrast, screening-based studies of apparently healthy cats have reported substantially higher prevalence rates, ranging from 14.7% to 37.4%, which can be attributed to broader diagnostic criteria, inclusion of subclinical cases, and differences in study design [18]. The lower prevalence observed in this referral-based cohort likely reflects a more selective population of clinically overt disease and highlights the importance of distinguishing between screening and diagnostic cohorts when interpreting epidemiological data. Regional differences in referral patterns, clinician experience, and diagnostic protocols may further contribute to variability among studies.

Breed-specific prevalence varied considerably in the present study. A high prevalence of hypertrophic cardiomyopathy was observed in Persian cats (54.8%) within this referral cohort. Historically high-risk breeds such as Maine Coons and Ragdolls have been shown to carry MYBPC3 mutations associated with familial hypertrophic cardiomyopathy [19]; however, direct comparison with these breeds should be interpreted cautiously in the present study. Persian cats are among the most common breeds in Tehran and were therefore disproportionately represented in the study population, whereas Maine Coons and Ragdolls were relatively uncommon. Consequently, the observed differences in prevalence likely reflect local breed popularity and referral patterns rather than definitive differences in genetic susceptibility. Although no genetic studies to date have specifically investigated hypertrophic cardiomyopathy in Persian cats, the high prevalence observed in this cohort underscores the need for targeted genetic research in regions where this breed is prevalent. At present, genetic testing for hypertrophic cardiomyopathy–associated mutations, including MYBPC3 variants, is not available in Iran, which limits confirmation of molecular diagnoses and hereditary mechanisms.

Elevated prevalence rates were also observed in Sphynx, British Shorthair, Domestic Shorthair, and Scottish Fold cats. These findings are likely influenced by the symptomatic and referral-based nature of the study population rather than representing definitive breed susceptibility, although a hereditary contribution cannot be excluded. Previous multicenter investigations, including the REVEAL study, have identified genetic predispositions in breeds such as the Sphynx and Ragdoll [20]. Variability in breed prevalence across studies may result from differences in breed distribution, referral practices, and diagnostic thresholds, emphasizing the importance of localized surveillance strategies that consider regional breed demographics and clinical referral behavior.

Echocardiographic findings in this study were consistent with established diagnostic features of hypertrophic cardiomyopathy. All affected cats demonstrated symmetric concentric left ventricular hypertrophy, while interventricular septal thickening and left atrial enlargement were identified in substantial subsets of cases. These findings align with previously reported data and reinforce the central role of echocardiography in disease diagnosis and staging [21, 22].

Univariate logistic regression analysis revealed that adult cats aged 5 to 9 years had significantly higher odds of hypertrophic cardiomyopathy diagnosis than senior cats older than 9 years (OR = 5.70; 95% CI: 3.18–10.23; *p* < 0.001). This observation is consistent with data derived from symptomatic populations, including the study by Atkins et al. (1992), which reported a peak prevalence at a mean age of 6.5 ± 4 years [17, 23]. In contrast, screening-based studies of asymptomatic cats, such as the CatScan project, have demonstrated increasing prevalence with advancing age, reaching 25% to 30% in cats older than nine years [17]. This discrepancy may be attributable to referral patterns and survival bias, as adult cats may be more likely to undergo diagnostic evaluation in clinical settings. Additionally, distinguishing pathological myocardial thickening from age-related myocardial changes remains a clinical challenge. As noted by Kittleson and Côté (2021), mild myocardial thickening may be observed from as early as 3 months to as late as 20 years of age [24]. Therefore, rigorous exclusion of secondary causes, including systemic hypertension and hyperthyroidism, as applied in the present study, is essential to ensure diagnostic accuracy [25].

A significant association was also identified between elevated body condition score and hypertrophic cardiomyopathy. Cats with a body condition score of 6 out of 9 or greater exhibited an increased likelihood of disease, with an odds ratio of 4.05 (95% CI: 2.29 to 7.17; *p* < 0.001). This finding is consistent with previous studies linking excess body weight to myocardial hypertrophy and altered metabolic profiles [26]. Although hypertrophic cardiomyopathy was also identified in cats with ideal body condition, the results support body condition score as a potentially modifiable cardiovascular risk factor. Together, age and body condition likely interact with underlying genetic susceptibility in the pathogenesis of feline hypertrophic cardiomyopathy.

Arterial thromboembolism emerged as a critical and life-limiting complication in affected cats [27]. Outcomes were generally poor, with most cases resulting in euthanasia or natural death, particularly among Domestic Shorthair and Persian cats. This observation aligns with existing literature identifying arterial thromboembolism as a frequent sequela of advanced hypertrophic cardiomyopathy and a major contributor to morbidity and mortality [24, 28]. Kaplan–Meier survival analysis demonstrated a marked decline in survival probability, from over 80% at diagnosis to approximately 20% by the end of the follow-up period, consistent with previously reported trends. The absence of standardized treatment protocols across participating hospitals may have influenced outcomes and limited comparability with prospective studies employing uniform anticoagulation or thrombolytic strategies.

Despite the inclusion of a large multicenter cohort, this study has several limitations related to its retrospective and referral-based design. Restriction of inclusion to cats evaluated for suspected cardiac disease limits generalizability and may underestimate the prevalence of subclinical hypertrophic cardiomyopathy. Echocardiographic assessments relied on contemporaneous clinical reports, and numerical measurements were not consistently available in a standardized format across centers, precluding quantitative analysis of individual echocardiographic variables. Allometric scaling of left ventricular wall thickness to body size was not performed, and the use of a fixed diagnostic cutoff may have resulted in misclassification in cats at the extremes of body size. Additionally, inter-institutional variability in diagnostic approaches, management strategies, and follow-up duration may have influenced outcome estimates. Finally, associations observed with age and body condition score should be interpreted as non-causal.

## Conclusion

This study highlights the prevalence, clinical features, and thromboembolic complications of hypertrophic cardiomyopathy in a referral population of cats evaluated for suspected cardiac disease in Tehran, Iran. With an observed clinical prevalence of 9.1% within this referral population, hypertrophic cardiomyopathy was particularly common in Persian cats, supporting the presence of breed-related predispositions. Key clinical findings included muffled heart sounds, gallop rhythms, and cardiomegaly. Arterial thromboembolism was associated with reduced survival, as demonstrated by declining survival probability in Kaplan–Meier analyses. Collectively, these findings emphasize the importance of early diagnosis, targeted screening strategies, and proactive clinical management to improve outcomes in feline populations at increased risk. Future prospective studies incorporating genetic analyses are warranted to further elucidate underlying disease mechanisms.

## Data Availability

The datasets analyzed during the current study are available from the corresponding author upon reasonable request. Individual medical records are not publicly available due to privacy considerations; however, aggregated data can be shared.
